# Colossal Seebeck effect enhanced by quasi-ballistic phonons dragging massive electrons in FeSb_2_

**DOI:** 10.1038/ncomms12732

**Published:** 2016-09-06

**Authors:** H. Takahashi, R. Okazaki, S. Ishiwata, H. Taniguchi, A. Okutani, M. Hagiwara, I. Terasaki

**Affiliations:** 1 Department of Applied Physics, The University of Tokyo, 7-3-1, Hongo, Bunkyo, Tokyo 113-8656, Japan; 2 Department of Physics, Nagoya University, Nagoya 464-8602, Japan; 3 Department of Physics, Faculty of Science and Technology, Tokyo University of Science, Noda 278-8510, Japan; 4 PRESTO, Japan Science and Technology Agency, Kawaguchi, Saitama 332-0012, Japan; 5 Center for Advanced High Magnetic Field Science, Graduate School of Science, Osaka University, 1-1 Machikaneyama, Toyonaka, Osaka 560-0043, Japan

## Abstract

Phonon transport is an essential property of thermoelectric materials. Although the phonon carries heat, which reduces the thermoelectric efficiency, it contributes positively to the Seebeck coefficient *S* through the phonon-drag effect, as typified by the high-purity semiconductors, which show fairly large *S* at cryogenic temperatures. Although such a large *S* is attractive in terms of Peltier cooling, a clear guiding principle for designing thermoelectric materials enriched by the phonon-drag effect remains to be established. Here we demonstrate that a correlated semiconductor, FeSb_2_, is a promising thermoelectric material featuring quasi-ballistic phonons dragging *d* electrons with large effective mass. By changing the sample size within the sub-millimetre order for high-purity single crystals, we succeed in substantially increasing *S* to as much as −27 mV K^−1^ at low temperatures. Our results exemplify a strategy for exploring phonon-drag-based thermoelectric materials, the performance of which can be maximized by combining heavy electrons with ballistic phonons.

## Abstract (web summary)

Thermoelectric performances depend on phonon and electron transport. Here, Takahashi *et al*. show that the large Seebeck coefficient observed in high-purity single-crystal FeSb_2_ is due to the phonon-drag effect and to the high effective mass of *d* electrons interacting with quasi-ballistic phonons.

## Graphical abstract

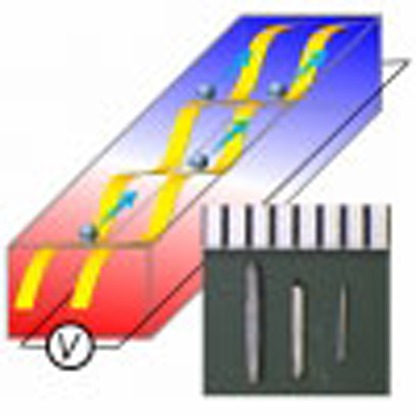

Recent progress in thermoelectric materials has been achieved primarily by understanding the phonon transport properties^1^,^2^,^3^,^4^. To improve the thermoelectric efficiency defined by the dimensionless figure of merit *zT*=*S*^2^*Tρ*^−1^*κ*^−1^, where *S*, *ρ*, *κ* and *T* are the Seebeck coefficient, electrical resistivity, thermal conductivity and absolute temperature, respectively^5^, reduction of the phonon thermal conductivity has been recognized as a key strategy. This concept is exemplified by nanostructuring techniques, which effectively reduces the mean free paths (MFPs) of the phonons^6^,^7^. On the other hand, phonons with long MFPs strongly affect *S* through the phonon-drag effect, which is a type of non-equilibrium phenomenon, that is, the phonon current drags the charge carriers, giving rise to the additional thermoelectric voltage^8^,^9^,^10^. The phonon-drag effect is proportional to the strength of the electron-phonon coupling and the relaxation time of the phonons coupled to electrons. To take advantage of the phonon-drag effect for thermoelectric applications, the magnitude of this effect has to be maximized, to overcome the disadvantage of *κ* enhancement caused by the long-MFP phonons. However, the development of phonon-drag-based thermoelectrics has been hampered by difficulty in describing the phonon-drag effect, as well as the absence of an ideal system for systematic investigations.

In the simple phenomenological model proposed by Herring^9^, the phonon-drag component of the Seebeck coefficient, *S*_ph_, is characterized by the relation, *S*_ph_∝*l*_ph_*μ*_el_^−1^, where *l*_ph_ and *μ*_el_ represent the phonon MFP and carrier(electron) mobility, respectively. This relation is consistent with the fact that a giant Seebeck effect has been reported for conventional high-purity semiconductors with ballistic phonons, the MFP of which is on the order of millimetres^8^,^9^,^10^. However, high thermoelectric efficiency has not been reported for conventional semiconductors because of the huge *κ*-values inherent in such long-MFP phonons^11^. Following the above discussion, high-purity correlated semiconductors promise to show an even higher thermoelectric efficiency, owing to the larger phonon-drag Seebeck effect, because the carrier mobility *μ* is inversely proportional to the effective mass m*, which has not been experimentally demonstrated.

To date, a colossal Seebeck coefficient for an intermetallic compound, FeSb_2_, (as large as |*S*|≃45 mV K^−1^) has been reported^12^,^13^. However, as far as we know, the reproducibility is questionable; thus, the origin of such a large Seebeck effect remains controversial^14^,^15^,^16^,^17^,^18^. There are two major explanations for its origin. One is based on the narrow gap structure formed by hybridization between localized Fe 3*d* electrons and itinerant Sb 5*p* electrons, as typified by Kondo insulators^19^,^20^,^21^,^22^,^23^. The other is based on the phonon-drag effect^17^,^24^,^25^. A recent first-principles study of FeSb_2_ predicts the presence of ballistic phonons with long MFPs on the order of 100 μm at low temperatures^26^. However, the former *d*–*p* hybridization gap scenario is not reproduced by band calculations considering the electron correlations^24^ and the latter phonon-drag scenario is inconsistent with the fact that the impurity concentration in the reported samples is higher than that of high-purity semiconductors^15^,^18^,^27^.

Here we show that FeSb_2_ is a canonical correlated semiconductor showing a colossal phonon-drag Seebeck effect, which features an exquisite combination of quasi-ballistic phonons and massive *d* electrons. Using transport measurements of high-purity single crystals with different dimensions, we identify the presence of nearly ballistic phonons that hugely enhance the Seebeck effect in FeSb_2_. In addition, the effective mass of charge carriers is found to be reasonably large by cyclotron resonance (CR) measurements at low temperatures.

## Results

### Crystal size effect on the transport properties

**Figure 1:**
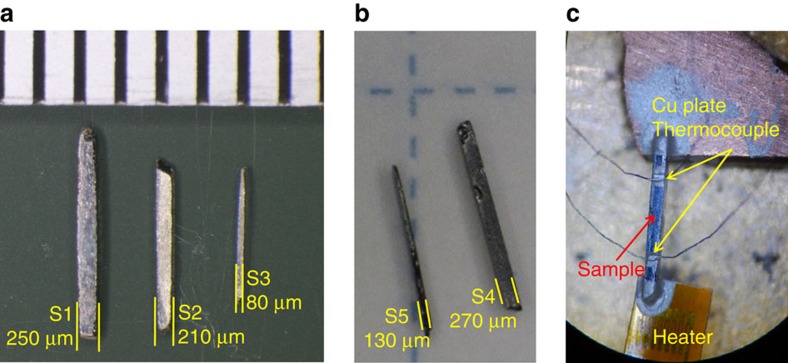
Sample dimensions and setup for measuring the Seebeck coefficient and thermal conductivity. (**a**) Sample dimensions of S1, S2 and S3, which have cross-sections of 250 × 245 μm^2^, 210 × 205 μm^2^ and 80 × 160 μm^2^, respectively. (**b**) Two samples, S4 and S5, with cross-sections of 270 × 250 μm^2^ and 130 × 150 μm^2^, respectively, are obtained by dividing a single crystal. (**c**) Measurement system for Seebeck coefficient *S* and thermal conductivity *κ*. The sample is attached to the heater and Cu plate by silver paste (DuPont 4922N), with thermocouples on both ends. We measure the thermoelectric voltage of chromel-constantan thermocouples to evaluate the temperature difference and thermoelectric voltage of the sample using chromel wires.

**Figure 2:**
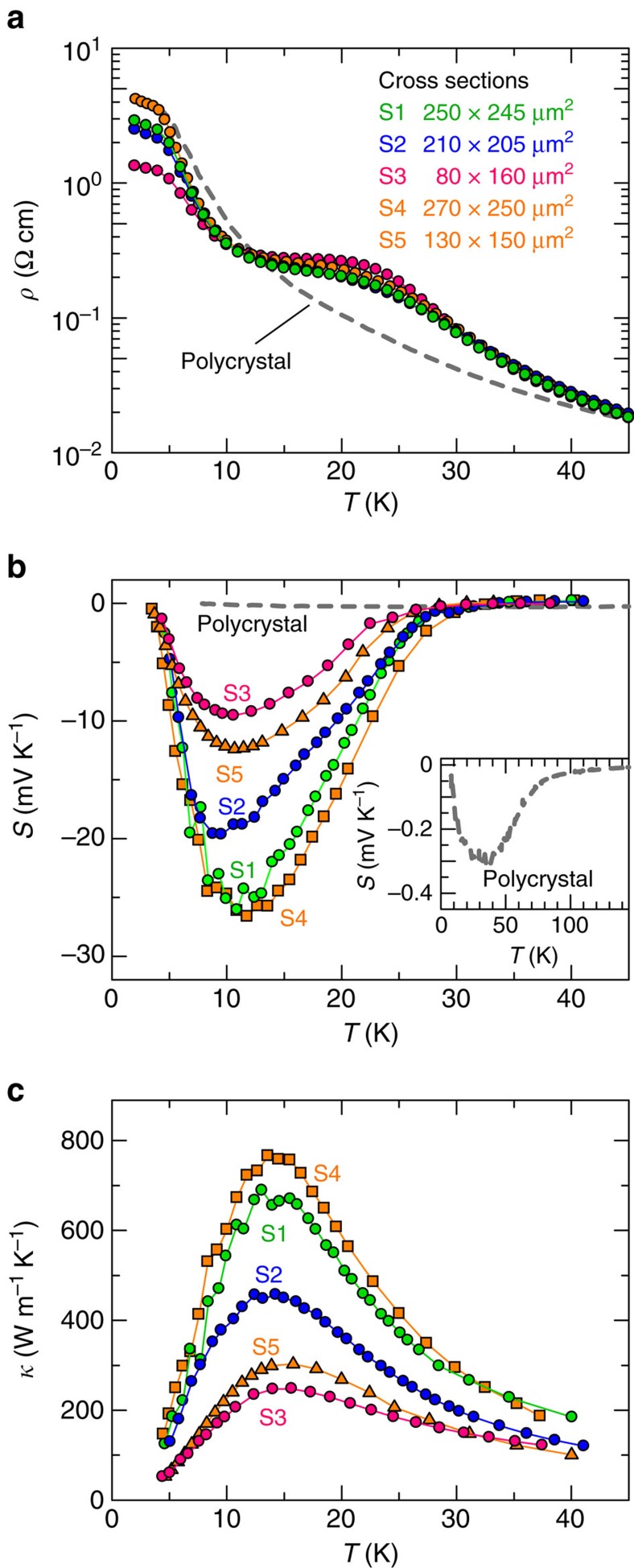
Sample size effect on the electrical and thermal transport properties. (**a**) Temperature dependence of electrical resistivity. (**b**,**c**) Temperature dependence of Seebeck coefficient and thermal conductivity. Both properties show a striking size dependence in the high-purity single crystals. *ρ* and *S* for the low-purity polycrystalline sample are plotted as grey lines in **a**,**b**, respectively.

Using high-purity single-crystalline samples of FeSb_2_ with different dimensions (80 × 160 μm^2^ ≤Sample cross-section ≤250 × 270 μm^2^), as shown in Fig. 1a,b, we studied the size effect on *ρ*, *S* and *κ*. Single crystals of FeSb_2_ reportedly exhibit two distinct types of transport properties, depending on the crystal growth conditions^16^: one shows semiconducting behaviour along all the axes, whereas the other shows semiconducting behaviour along the *a*- and *b*-axes but metallic behaviour along the *c*-axis above 50 K. Thus, to discuss the intrinsic size effect on the thermoelectric properties, we checked the crystallographic orientation dependence of *ρ* and found that our samples are nearly isotropic semiconductors (see Supplementary Figs 1 and 2). The crystallographic orientations of samples S1–S3 were characterized by Laue diffraction, as shown in Supplementary Fig. 3. Furthermore, to completely exclude the extrinsic effects arising from differences in the sample quality and crystallographic orientation, we prepared two samples, S4 and S5, which were obtained by cutting a single crystal into two pieces (see Supplementary Fig. 4). To determine *S* and *κ*, we used a steady-state technique, as shown in Fig. 1c, where two chromel-constantan thermocouples were mounted on the sample to measure the temperature difference and thermoelectric voltage. Figure 2a shows the temperature dependence of *ρ*; all values of *ρ* are almost the same, so it is essentially independent of the sample dimensions (the temperature dependence of *ρ* above 45 K is shown in Supplementary Fig. 5). From the magnetotransport properties, we estimated the carrier concentration *n* and mobility *μ* of all the samples from 8 to 30 K (see Supplementary Figs 6–8 and Supplementary Note 1). The obtained parameters are nearly invariant for all the samples with different dimensions, indicating that they share essentially the same electronic states with slightly different impurity levels.

In Fig. 2b,c, we plot *S* and *κ* as a function of temperature, revealing a significant sample-size effect. S4 has a considerably large |*S*| value of 27 mV K^−1^ at 10 K and the maximum value decreases to 10 mV K^−1^ (S3) with decreasing sample cross-section. As the electrical transport properties (*ρ*, *n*, and *μ*) are exactly the same in S4 and S5, it should be the sample size rather than the electronic conduction that plays a major role in the striking enhancement of *S*. Corresponding to the size dependence of *S*, the maximum value of *κ* also decreases from 770 W m^−1^ K^−1^ (S4) to 230 W m^−1^ K^−1^ (S3) at 15 K. The electronic component of *κ*, evaluated using *ρ* and the Wiedemann–Franz law, was found to be negligible for each sample, indicating that the size effect on *κ* stems from phonon transport, which is, therefore, likely to be related to the Seebeck effect. This idea is further supported by the fact that the |*S*| value for a low-purity (99.9% purity) polycrystalline sample (∼300 μV K^−1^) is two orders of magnitude smaller than that for the high-purity (99.999% purity) single crystals (Fig. 2b, inset), whereas the *ρ*-values of both samples at low temperatures are comparable (Fig. 2a). These results indicate that phonon scattering by crystal/grain boundaries and impurities significantly affects on the Seebeck effect^27^.

### Surface scattering effect on the phonon MFP

**Figure 3:**
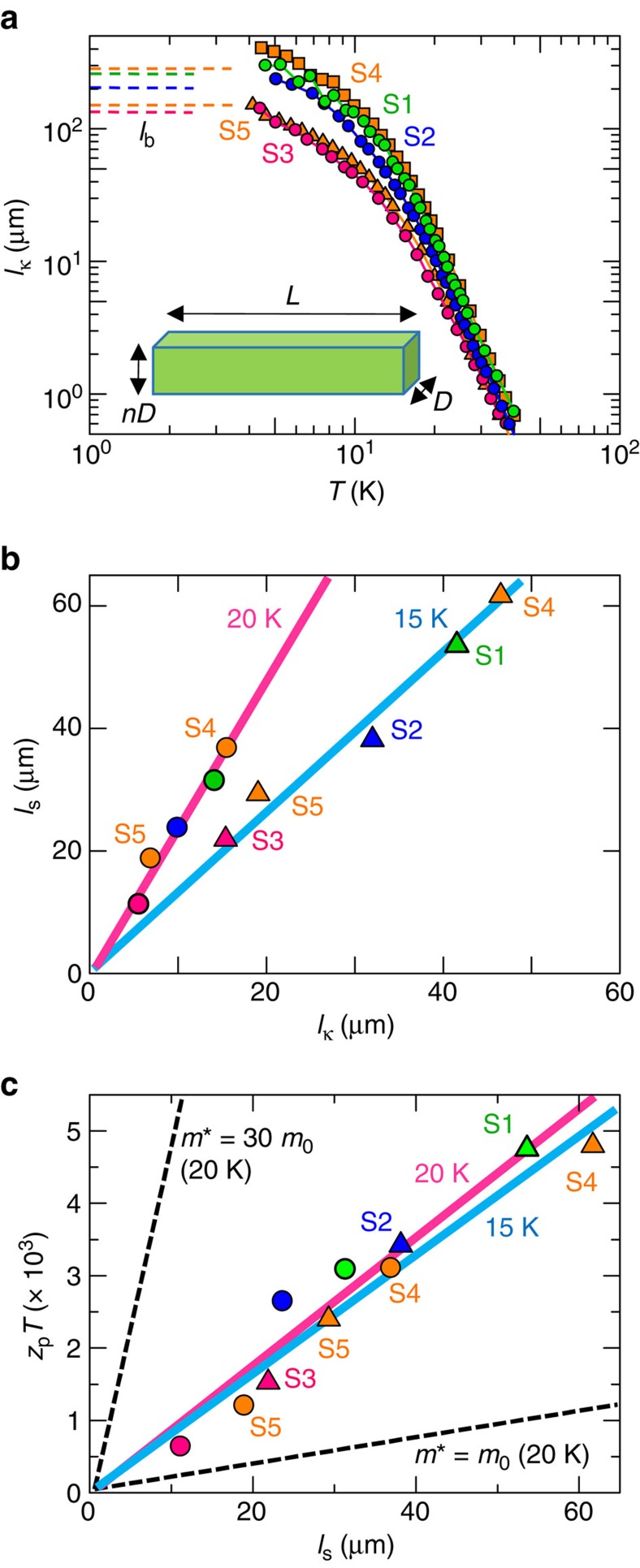
Phonon MFP and thermoelectric figure of merit for the phonon-drag effect. (**a**) Temperature dependence of the phonon MFP, *l*_κ_, evaluated from the thermal conductivity with Fourier's law. Broken lines are phonon MFP values (*l*_b_ described by the equation (1)) evaluated by considering scattering at crystal boundaries^29^. Inset shows the sample shape with length scales used for equation (1). (**b**) Comparison of thermal (*l*_κ_) and phonon-drag (*l*_s_) phonon MFPs at 15 K (triangles) and 20 K (circles)^9^. (**c**) Phonon-drag dimensionless figure of merit *z*_p_*T* with respect to *l*_s_ at 15 K (blue line) and 20 K (red line). *z*_p_*T* also depends on the electron effective mass m* as shown by the broken lines.

Next, we evaluated the phonon MFPs using Fourier's law (*κ*=⅓*Cvl*_κ_) with the Debye model (Debye temperature=340 K), where *C*, *v* and *l*_κ_ are the specific heat, phonon velocity (*v*=3,100 m s^−1^) and MFP of the phonon involved in the thermal conductivity, respectively^12^. Here we assume that the phonon transport is isotropic, as it has been reported that the phonon velocity and thermal conductivity are almost independent of the crystal orientations^12^,^28^. As shown in Fig. 3a, *l*_κ_ increases to on the order of 100 μm with decreasing temperature, suggesting that *l*_κ_ is dominated by crystal-boundary scattering at low temperature. Here we define *l*_b_ as the MFP dominated by such crystal boundary scattering. Provided that the sample surface with an appropriate roughness acts as diffuse scatterers of phonons, *l*_b_ for the rectangular sample with side dimensions *D* and *nD* (Fig. 3a) can be evaluated as^29^


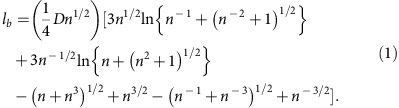


By using this equation, the *l*_b_ value for samples S1, S2, S3, S4 and S5 were estimated to be 280, 230, 120, 290 and 150 μm, respectively. This estimation method loses its validity when the length scale of the rectangular sample, *L*, is comparable to the phonon MFP^30^, whereas this method is commonly used when *L* is much larger than the phonon MFP, which is in the order of *D* (ref. 29). This situation (*L*>>*D*∼*l*_b_) is satisfied in our experiment and, remarkably, the experimental data approach the evaluated values with equation (1) (dotted lines in Fig. 3a) at low temperature, indicating that boundary scattering dominates the thermal conductivity *κ*.

### Phonon-drag effect

To clarify the relation between the phonon transport and the Seebeck effect, we quantitatively analyse *S* assuming the phonon-drag effect. In the approximate expression of the theory proposed by Herring, the phonon-drag component of *S* equals *βvl*_s_*μ*^−1^*T*^−1^, where *l*_s_ is the MFP of the phonon involved in the phonon-drag effect and *β* is a parameter between 0 and 1 characterizing the relative strength of electron–phonon interactions^9^. If electrons are scattered only by phonons, *β* is 1, but additional scattering processes, such as impurity scattering, can bring it to 0 (ref. 9). From 30 to 15 K, *μ* increases with decreasing the temperature with the slope of *T*^−3/2^ (see Supplementary Fig. 8b), which is typically observed in the semiconductors, when the electrons mainly scattered by phonon. In addition, the increment of |*S*| is also observed in the same temperature range. These results imply that the carriers are primarily scattered by phonons. Therefore, we adopt the value *β*=1 for the evaluation of *l*_s_ at 20 and 15 K. Here the electronic part of *S* is removed to evaluate *l*_s_ at 15 and 20 K, which contributes <20% of the total values (see Supplementary Fig. 9 and Supplementary Note 2). Below 10 K, *l*_s_ cannot be precisely evaluated, because |*S*(*T*)| is diminished, suggesting that *β* diverges from unity.

Here we compare the length scales of the thermal (*l*_κ_) and phonon-drag (*l*_s_) phonon MFPs, as shown in Fig. 3b. The values of *l*_κ_ and *l*_s_ have the same order of magnitude and show a clear proportional relationship. Although the phonon MFPs are distributed in a certain range at finite temperature, the linear relationship between *l*_s_ and *l*_κ_ strongly suggests that it is the crystal-boundary scattering that primarily determines the lengths of *l*_s_ and *l*_κ_ with a small wave vector (the phonon MFP is order of 100 μm). In other words, the similar size effect observed for *l*_κ_ and *l*_s_ indicates that the phonons, of which MFP is shortened with decreasing sample dimensions, contribute significantly to the colossal *S* value as well as *κ*. This result is the first direct evidence that the colossal *S* value in FeSb_2_ stems from the large enhancement of the phonon-drag effect by the presence of the nearly ballistic phonons.

### CR measurements

**Figure 4:**
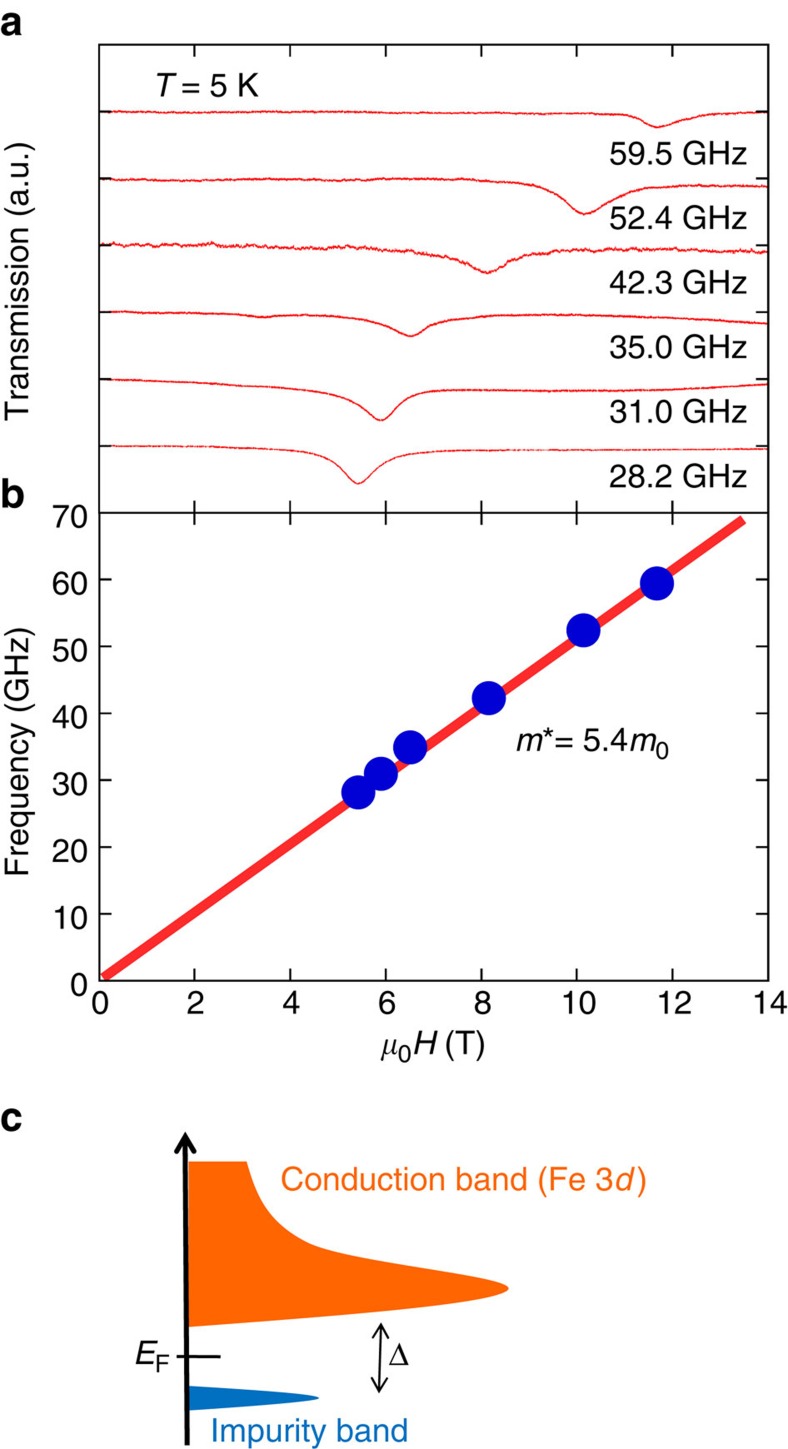
CR measurement. (**a**) CR spectra at 5 K at the designated frequencies. (**b**) Frequency versus magnetic field plot of the CR fields obtained from the data in **a**. Straight line gives a cyclotron effective mass m* of 5.4*m*_0_ (where *m*_0_ is the bare electron mass). (**c**) Band diagram of FeSb_2_; a small energy gap Δ appears between the conduction and impurity bands^15^,^18^,^27^. The conduction band may possess a steep band edge associated with the localized Fe 3*d* orbitals, leading to the large electron mass.

According to the phonon-drag picture (*S*∝*l*_s_*μ*^−1^), the origin of the colossal *S* value of FeSb_2_ is ascribable not only to the nearly ballistic phonons but to the reduced electron mobility *μ*. As the *l*_s_ value in FeSb_2_ is two orders of magnitude shorter than that in Si (∼1 mm), the *μ*-value of FeSb_2_ should be small enough that the *S* value is comparable to or even larger than that of Si (ref. 10). Indeed, the *μ*-value of FeSb_2_ is reported to be much smaller (∼5 × 10^3^ cm^2^ V^−1^ s^−1^ at 15 K) than that of high-purity Si (∼2 × 10^5^ cm^2^ V^−1^ s^−1^ at 15 K)^15^,^31^. In the Drude model, *μ* is expressed as *eτ*_e_m^*−1^, where *τ*_e_ is the relaxation time of carriers. To see whether the *μ*-value of FeSb_2_ is reduced by the enhanced electron mass, we measured the CR for a high-purity single crystal of FeSb_2_ at 5 K. As shown in Fig. 4a, the resonance peak shifts to higher fields as *ν* increases. The linear fit of the *ν* dependence of the resonant frequency gives m*=5.4*m*_0_ (Fig. 4b), which is 20 times larger than that of Si (∼0.3*m*_0_)^31^.

## Discussion

The significantly large effective mass of FeSb_2_ indicates that the low mobility *μ* (∼*τ*_e_m^*−1^) reflects the large effective mass, as *τ*_e_ of FeSb_2_ is comparable to that of Si. Therefore, we attribute the origin of the colossal Seebeck effect in FeSb_2_ to the huge enhancement of the phonon-drag effect by the combination of the fairly large *l*_s_ (although it is not as large as that of high-purity Si) and the small *μ* inherent in the large m*. The large *l*_s_ is anticipated in the first principles study by Liao *et al*.^26^, which found that the phonon MFPs at 20 K are concentrated in the range of 1–200 μm when the phonons are not scattered by the impurities and crystal boundaries. In our study, we have successfully prepared the high-purity single crystals with the large dimensions by a flux method using the high-purity metal powders (see Methods), resulting in the fairly large *l*_s_. On the other hand, the large value of m* presumably reflects the characteristic band structure as predicted by thermodynamic measurements and band calculations^14^,^24^. It has been reported that there exists a high and steep density of states just above the Fermi level in the conduction band, which mainly consists of the Fe 3*d* orbitals (Fig. 4c).

Our result demonstrates a simple strategy to design phonon-drag-based thermoelectric materials. We depict the thermoelectric efficiency associated with the phonon-drag effect in Fig. 3c. The linear relation between *l*_s_ and *l*_κ_ (*l*_s_=*γl*_κ_) yields a simple expression for the phonon drag's dimensionless figure of merit *z*_p_*T*=*S*_ph_^2^*σTκ*^−1^=*Al*_s_*nμ*^−1^*T*^−1^ (*σ*=*neμ*, *A*=3*β*^2^*evC*^−1^*γ*^−1^), which can be improved by increasing *l*_s_ and m*. Whereas *z*_p_*T* is restricted to a low value (<1/4) for conventional semiconductors^11^, strongly correlated semiconductors such as Kondo insulators have the potential to greatly improve their efficiency because of their fairly large m* values. Therefore, provided that m* is as large as 1,000*m*_0_, as observed in heavy fermion systems^32^, and the phonon MFP is on the order of sub-millimetre, *z*_p_*T* is expected to reach 1, even at low temperatures. In such strongly correlated systems, the strong electron–electron scattering creates the quasi-particle with the larger effective mass compared with the band mass. Thus, the interaction between these quasi-particles and the phonons may cause a significant phonon-drag effect beyond the result of FeSb_2_.

## Methods

### Sample preparation

Single crystals of FeSb_2_ were grown by a self-flux method using metal powders of 99.999% (5N) pure Fe and 99.9999% (6N) pure Sb in an evacuated silica tube, as described in ref. 27. The single-crystalline nature was verified by Laue-pattern analysis (see Supplementary Figs 3 and 4). We measured the powder X-ray diffraction pattern of our samples and found no impurity peaks (see Supplementary Fig. 10). All samples were prepared simultaneously and then rubbed with sandpaper to yield five samples with different dimensions as shown in Fig. 1a. The prepared samples each have cross-section *F* and length *L* as shown in Table 1. S4 and S5 were obtained by cutting a single crystal into two pieces, meaning that their quality and crystallographic orientation should be identical. As all samples were ground by the same sandpaper, their surface conditions are expected to be almost the same. A polycrystalline sample was prepared by solid-state reaction using metal powders of 99.9% (3N) pure Fe and 99.9% (3N) pure Sb in an evacuated silica tube, as described in ref. 14.

### Transport measurement

The electrical resistivity *ρ* was measured as a function of temperature by a four-probe method.

### CR measurements

The CR was measured at 5 K from 28.2 to 59.5 GHz. The CR apparatus consists of a 16 T superconducting magnet (14 T at 4.2 K and 16 T at 2.2 K, Oxford Instruments, UK), a vector network analyser (AB Millimeter Co. Ltd, France) and a home-built transmission-type CR cryostat (usually used for electron spin resonance). With this vector network analyser, we can detect not only the amplitude of the CR signal but also its phase. A variable temperature insert with an inner diameter of 37 mm is plugged into the bore of the magnet; thus, the temperature can be varied from 1.5 to 200 K.

### Data availability

The data that support the findings of this study are available from the corresponding author upon request.

## Additional information

**How to cite this article:** Takahashi, H. *et al*. Colossal Seebeck effect enhanced by quasi-ballistic phonons dragging massive electrons in FeSb_2_. *Nat. Commun.* 7:12732 doi: 10.1038/ncomms12732 (2016).

## Supplementary Material

Supplementary Information Supplementary Figures 1-10, Supplementary Notes 1-2 and Supplementary References

## Figures and Tables

**Table 1:** Sample dimensions.

**Sample**	**Cross-section** ***F***	**Length** ***L***
S1	250 × 245 μm^2^	2.5 mm
S2	210 × 205 μm^2^	2.3 mm
S3	80 × 160 μm^2^	1.8 mm
S4	270 × 250 μm^2^	3.3 mm
S5	130 × 150 μm^2^	2.9 mm
