# Supplementary material for: Colossal Seebeck effect enhanced by quasi-ballistic phonons dragging massive electrons in FeSb2
Source: Nat Commun. 2016 Sep 6;7:12732. doi: 10.1038/ncomms12732 (PMC5025859; doi:10.1038/ncomms12732)
Supplement: Supplementary Information — Supplementary Figures 1-10, Supplementary Notes 1-2 and Supplementary References [file ncomms12732-s1.pdf]

Supplementary Figures

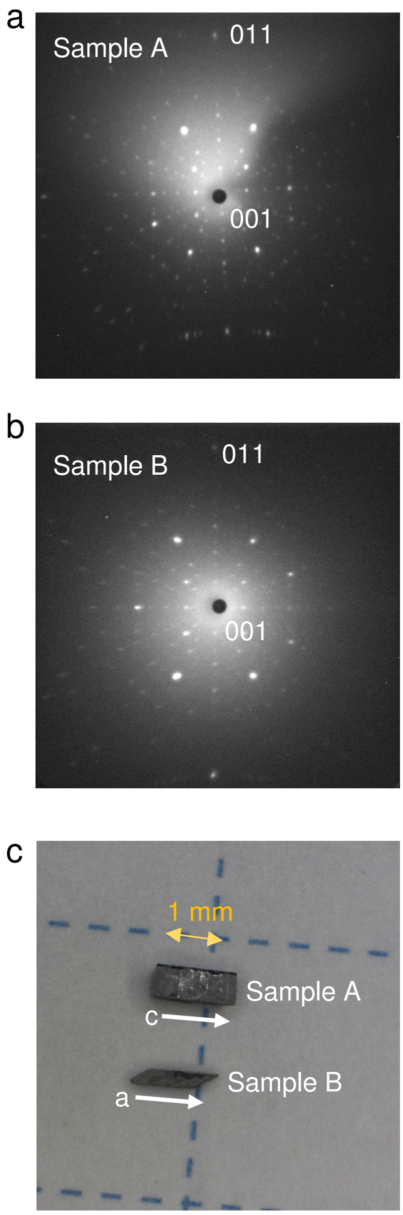

Supplementary Figure 1: **Laue patterns and picture of the single crystals.** (**a,b**) Laue patterns of sample A (**a**) and sample B (**b**). (**c**) Picture of the single crystals (sample A and sample B), whose crystallographic orientations are shown with white arrows.

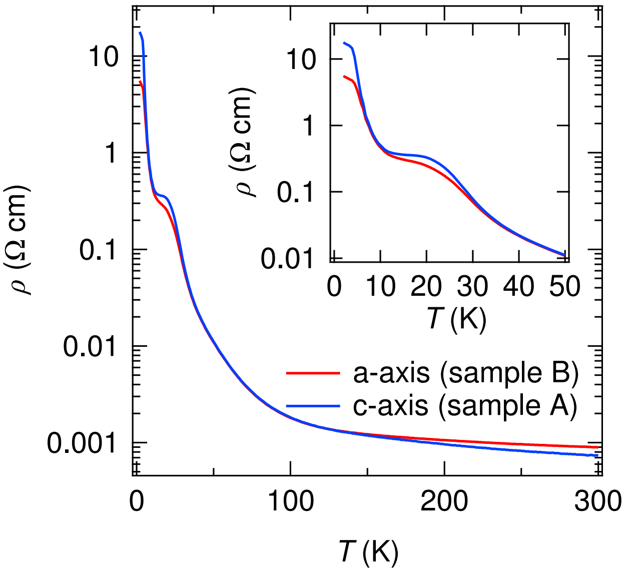

Supplementary Figure 2: **Temperature dependence of the electrical resistivity along the *c*-axis (sample A) and *a*-axis (sample B).**

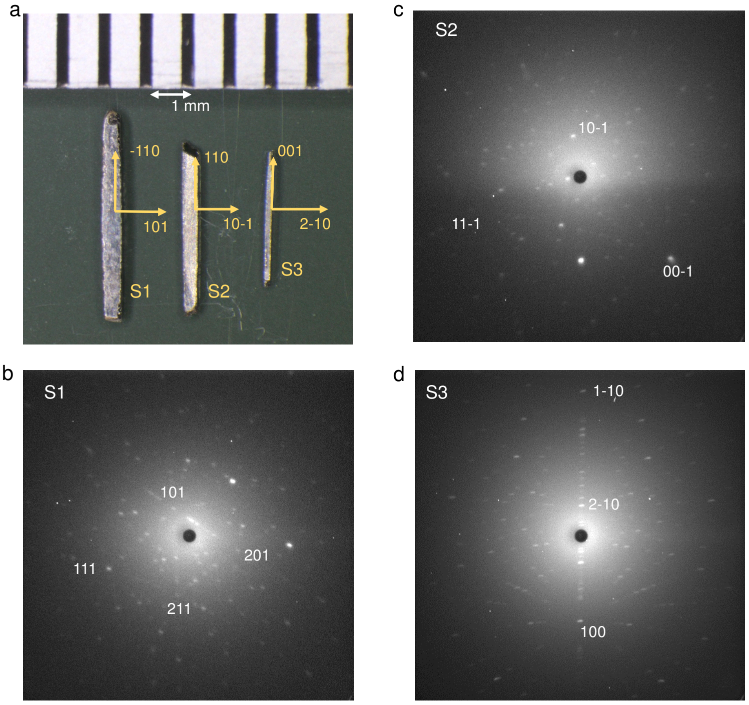

Supplementary Figure 3: **Sample picture and Laue patterns.** (**a-d**) Sample picture and Laue patterns of samples S1 (sample cross section $F = 250 \times 245$ μm^2), S2 ($F = 210 \times 205$ μm^2), and S3 ($F = 80 \times 160$ μm^2). From these Laue patterns, we identify the crystallographic orientation of the crystal bars. Transport properties were measured in (a,b)-plane for S1 and S2 and along c-axis for S3.

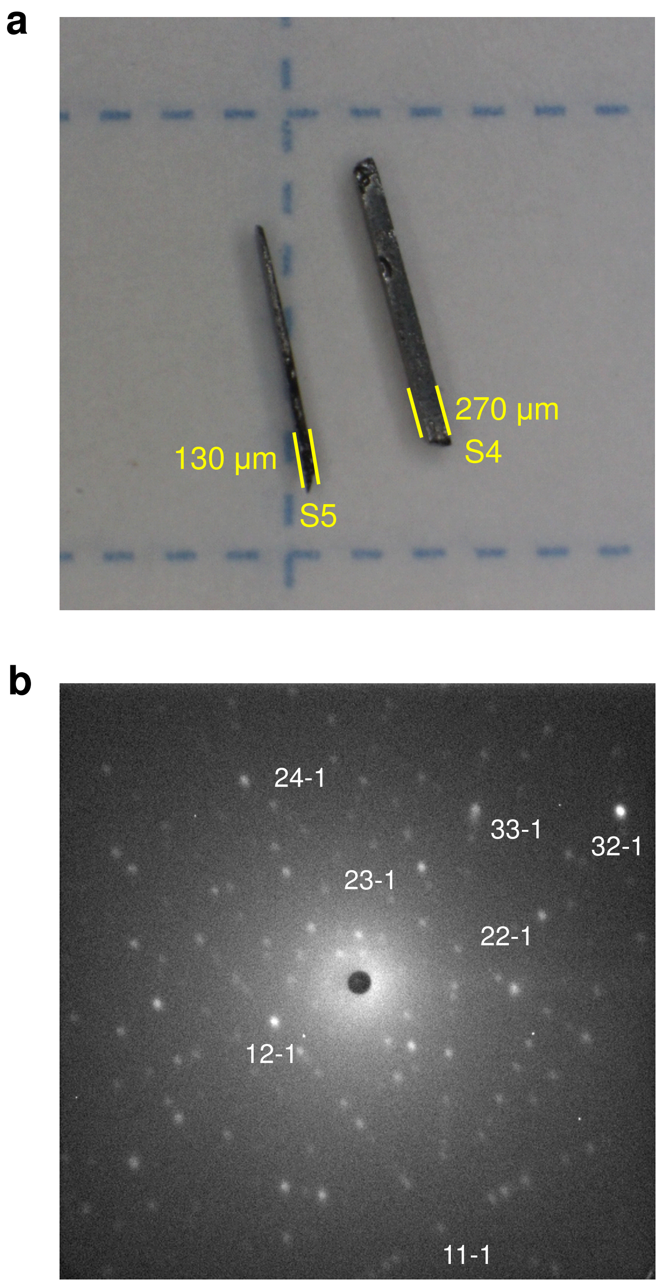

Supplementary Figure 4: **Sample picture and Laue patterns.** (**a,b**) Sample picture and Laue pattern of samples S4 ($F = 270 \times 250$ μm^2) and S5 ($F = 130 \times 160$ μm^2), which were prepared by cutting a single crystal into two pieces. The sample picture and Laue pattern are taken from one side of the single crystal.

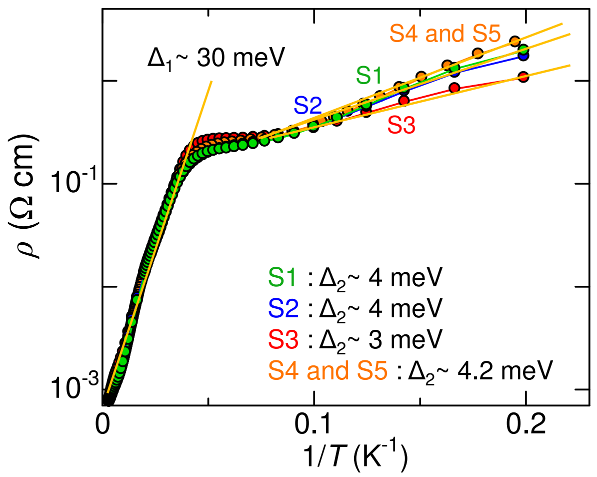

Supplementary Figure 5: **Electrical resistivity as a function of 1/*T*.** Two energy gaps above 30 K (Δ_1 ~ 30 meV) and below 10 K (Δ_2 ~ 4 meV for S1 and S2, ~ 3 meV for S3, and ~ 4.2 meV for S4 and S5) were evaluated using the thermally activated function $\rho = \rho_0 \exp(\Delta 2 k_B^{-1} T^{-1})$ as plotted by yellow lines. The previous studies of impurity effects and magnetotransport properties suggest that Δ_1 is the gap between the conduction and valence bands formed by Fe 3d orbitals, and Δ_2 is the gap between the conduction and impurity bands.[1–3]

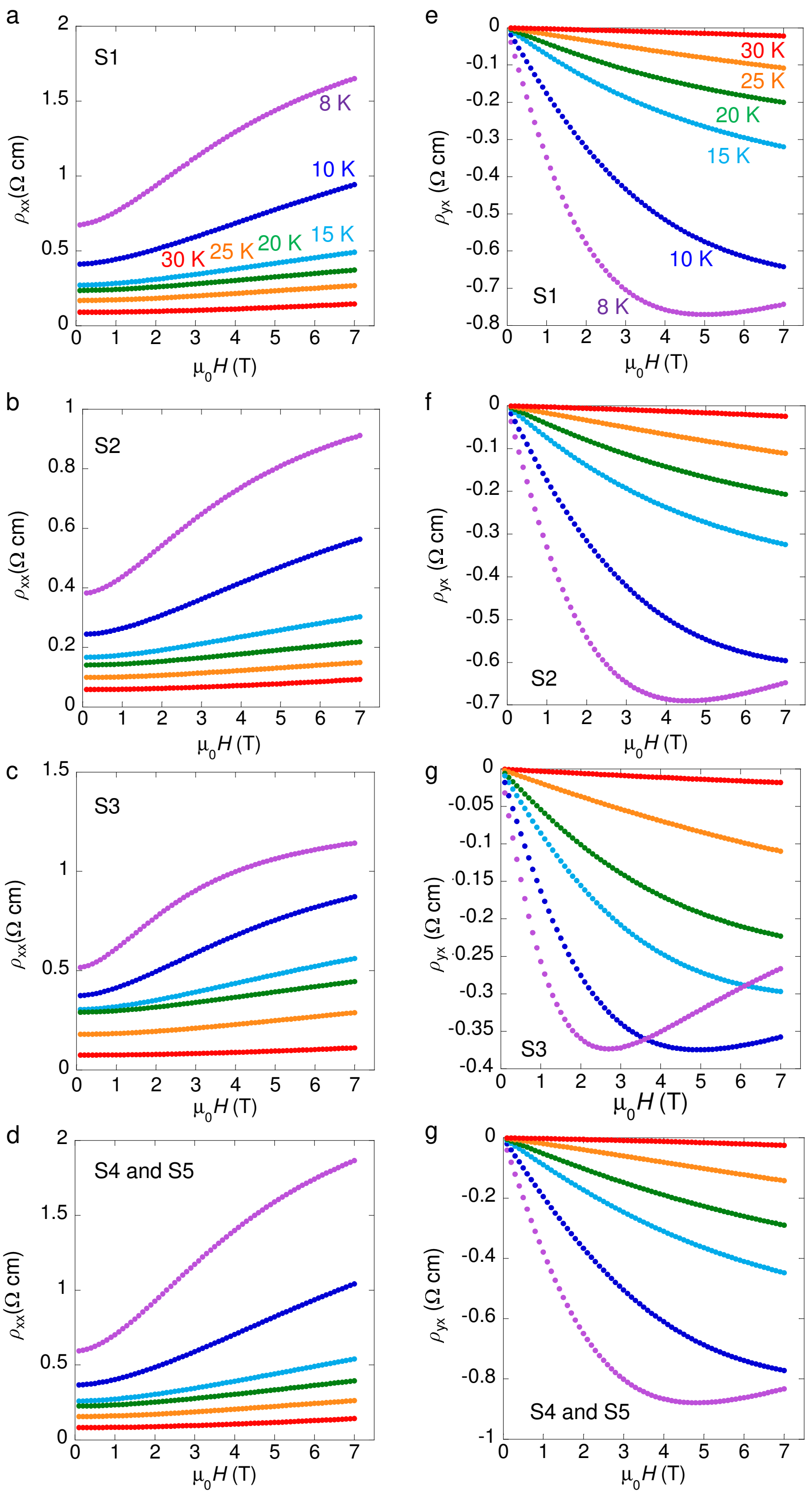

Supplementary Figure 6: **Magnetic field dependence of the resistivity tensors ρ_{xx} and ρ_{yx}.** (**a-d**) Magnetic field dependence of the resistivity tensor ρ_{xx} of S1, S2, S3, and S4(and S5). (**e-f**) Magnetic field dependence of the resistivity tensor ρ_{xy} of S1, S2, S3, and S4(and S5).

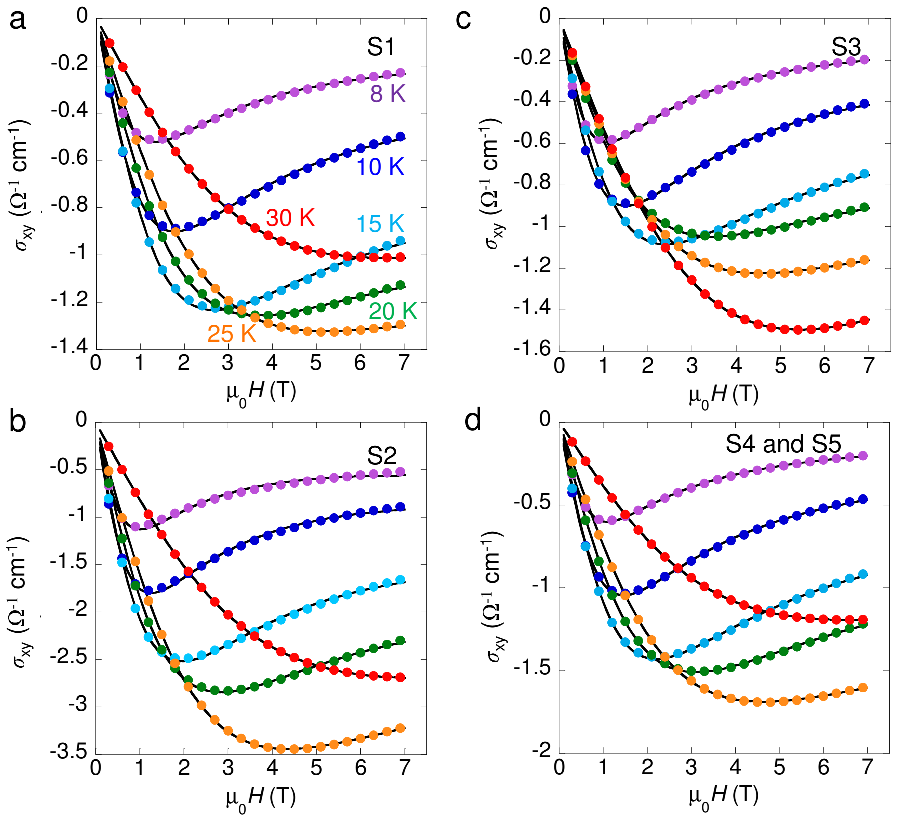

Supplementary Figure 7: **Magnetic field dependence of the conductivity tensor σ_{xy}.** (**a-d**) Magnetic field dependence of the conductivity tensor σ_{xy} of S1, S2, S3, and S4(and S5). Solid curves are the calculation using Supplementary Eq. 1 (see Supplementary Note 1).

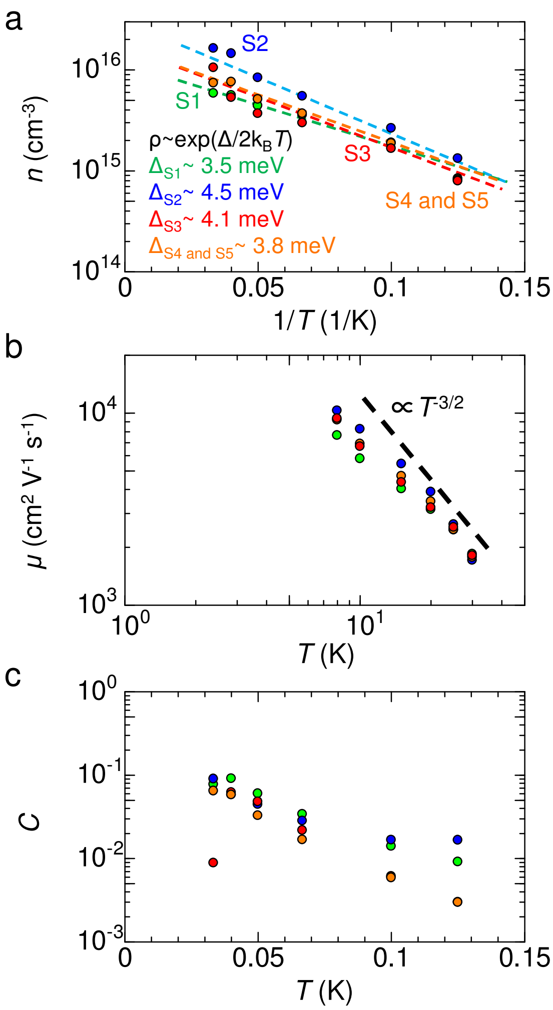

Supplementary Figure 8: **Temperature dependence of the physical parameters.** (**a-c**) Temperature dependence of the carrier concentration n, mobility μ, and low mobility component C evaluated from the fitting results shown in Supplementary Fig. 7. The energy gap Δ is evaluated from n (T) using the activation function as shown by dotted lines in Supplementary Fig. 8a. The slight size dependence of the energy gaps (3.5meV $< \Delta_2 <$ 4.5 meV) represents that the electronic states of all samples are almost identical: the concentration and/or level of the impurity states are slightly different. Since the analysis of n can exclude the contribution of the mobility, the energy gaps evaluated with this method are more precise compared with those evaluated from the electrical resistivity as shown in Supplementary Fig. 5. n and μ are almost independent of the crystal size and crystallographic orientation. μ increases with decreasing the temperature with the slope of $T^{-3/2}$, suggesting that the electrons mainly scattered by phonons. The contributions of the low-mobility components C to σ_{xy} are much smaller (less than 10 %) than those of the high-mobility carriers, and thus the high mobility carriers are predominant to the transport properties.

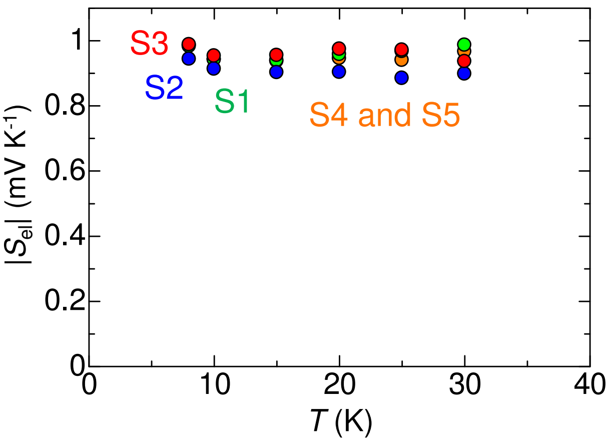

Supplementary Figure 9: **Seebeck coefficient of the electron-diffusion part.** Seebeck coefficient calculated from the Supplementary Eq. 2 (see Supplementary Note 2).

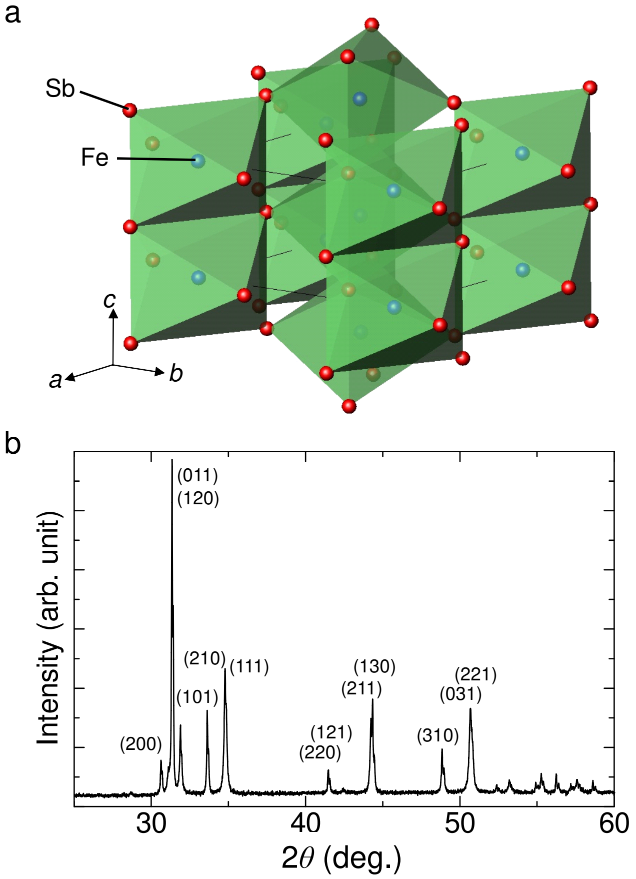

Supplementary Figure 10: **Crystal structure and powder X-ray diffraction pattern.** (**a,b**) Crystal structure and powder X-ray diffraction pattern of pulverized single crystals of $FeSb_2$ after removing Sb-flux with nitric acid. Crystal structure of $FeSb_2$ is the marcasite-type FeS_2 structure with the space group *Pnnm* (a = 5.83275, b = 6.5334, c = 3.1963).

Supplementary Notes

Supplementary Note 1: Sample dependence of the electric transport properties

The electronic states below 30 K can be precisely analyzed from the magnetotransport properties.[1] As shown in Ref. 1, the carrier concentration n and mobility μ are evaluated from the magnetic field H dependence of the conductivity tensor $\sigma_{xy}[= \rho_{yx}/(\rho_{xx}^2 + \rho_{yx}^2)]$ with a two-carrier model described as

$$\sigma_{xy}(H) = n_{xy} e \mu_{xy}^2 H \left[\frac{1}{1 + (\mu_{xy} H)^2} + C \right], \quad (1)$$

in which one carrier is of high mobility and the other is of low mobility ($\mu H \ll 1$). Here, C is the low-mobility component. Supplementary Fig. 6 shows the H dependence of the resistivity tensors ρ_{xx} and ρ_{yx}. The best agreements of σ_{xy} with Supplementary Eq. 1 is shown by black solid curves in Supplementary Fig. 7.

Supplementary Note 2: Seebeck coefficient of the electronic part

Here, we discuss the Seebeck coefficient of the electron-diffusion part. Since this compound shows the clear energy gap and small carrier concentration, the Seebeck coefficient can be calculated with a nondegenerate model expressed as[4]

$$S_{\rm el} = \pm \frac{k_B}{e} [\eta - (r + 5/2)]. \quad (2)$$

Here, η is the reduced Fermi energy written as $n = 2(\frac{2\pi m^* k_B T}{h^2})^{3/2} \exp\eta$, and r is a scattering parameter. In this calculation, we use n evaluated from σ_{xy}, $r = 3/2$, and $m^* = 5.4 m_0$ (m_0 is the bare electron mass), which is estimated from the cyclotron resonance experiment. Supplementary Fig. 9 plots the temperature dependence of the Seebeck coefficient evaluated from Supplementary Eq. 2. The Seebeck coefficient is almost 1 mV/K from 8 to 30 K, which is much smaller than that of the experimental results. This result indicates that the electron-diffusion part of the Seebeck coefficient plays a minor role in our compounds.

Supplementary References

1. Takahashi, H. *et al.* Low-temperature magnetotransport of the narrow-gap semiconductor $FeSb_2$. *Phys. Rev. B* **84,** 205215 (2011).

2. Takahashi, H. *et al.* Origin of the energy gap in the narrow-gap semiconductor $FeSb_2$ revealed by high-pressure magnetotransport measurements. *Phys. Rev. B* **88,** 165205 (2013).

3. Takahashi, H. *et al*. Effects of ppm-level imperfection on the transport properties of $FeSb_2$ single crystals. *J. Phys. Soc. Jpn.* **80,** 054708 (2011).

4. Sun, P. *et al*. $FeSb_2$: Prototype of huge electron-diffusion thermoelectricity. *Phys. Rev. B* **79,** 153308 (2009).

5. Petrovic, C. *et al*. Anisotropy and large magnetoresistance in the narrow-gap semiconductor $FeSb_2$ *Phys. Rev. B* **67,** 155205 (2003).

6. Jie, Q. *et al*. Electronic thermoelectric power factor and metal-insulator transition in $FeSb_2$. *Phys. Rev. B* **86,** 115121 (2012).
